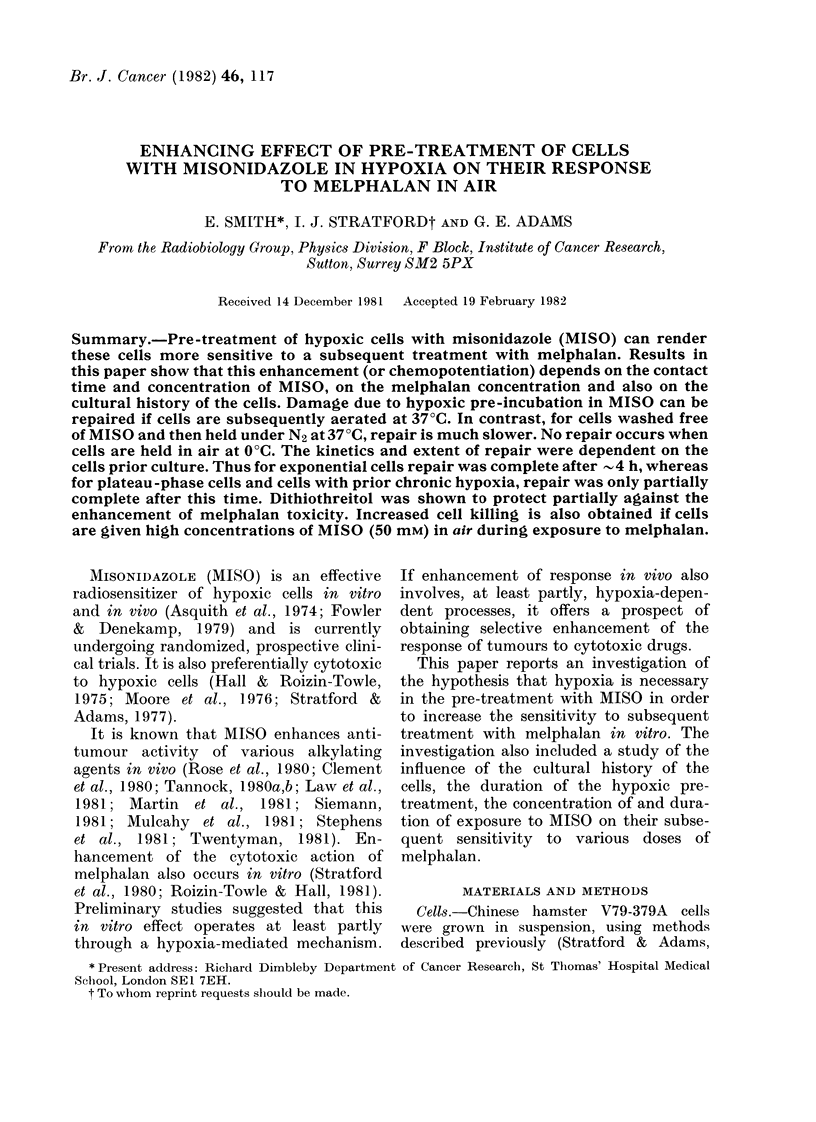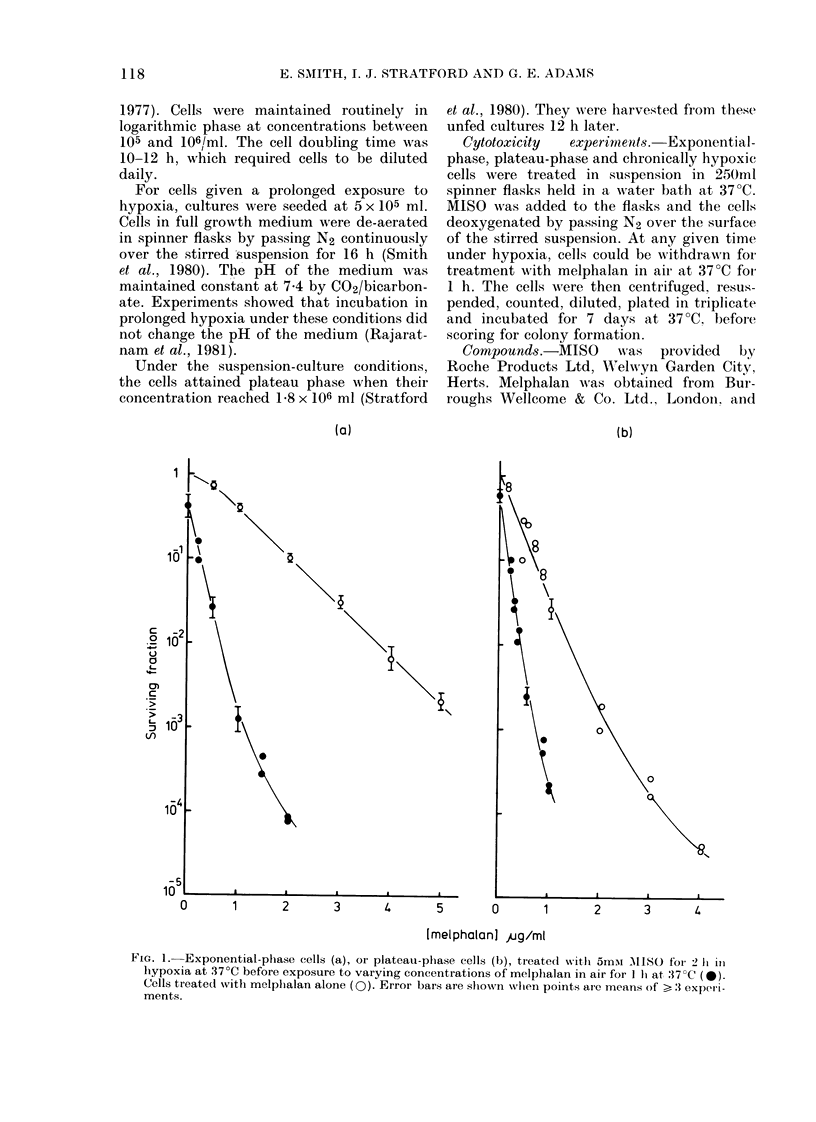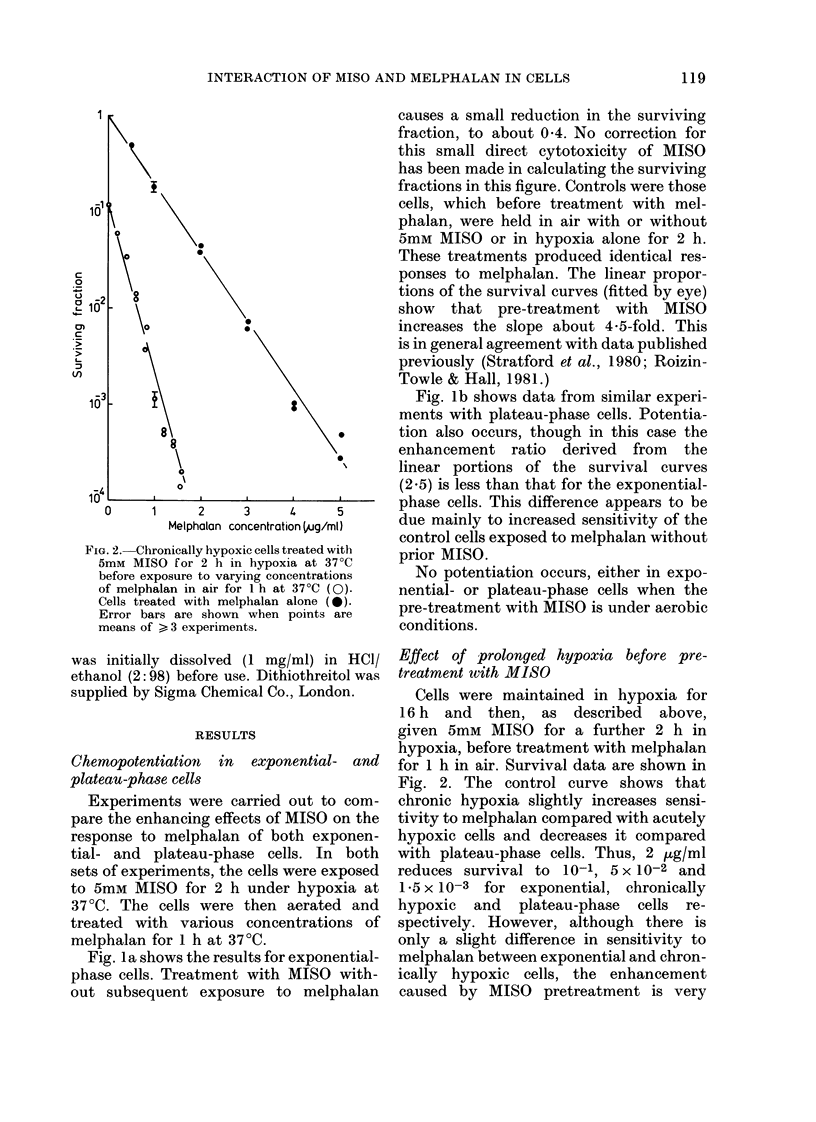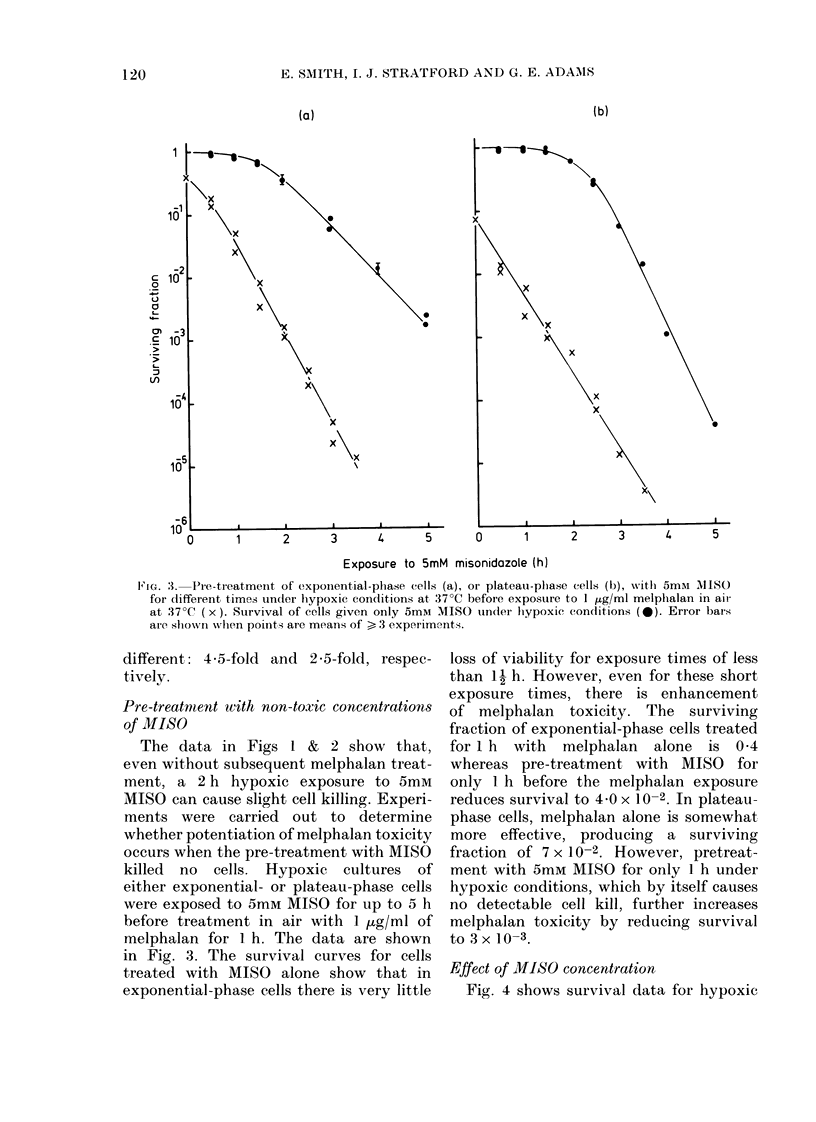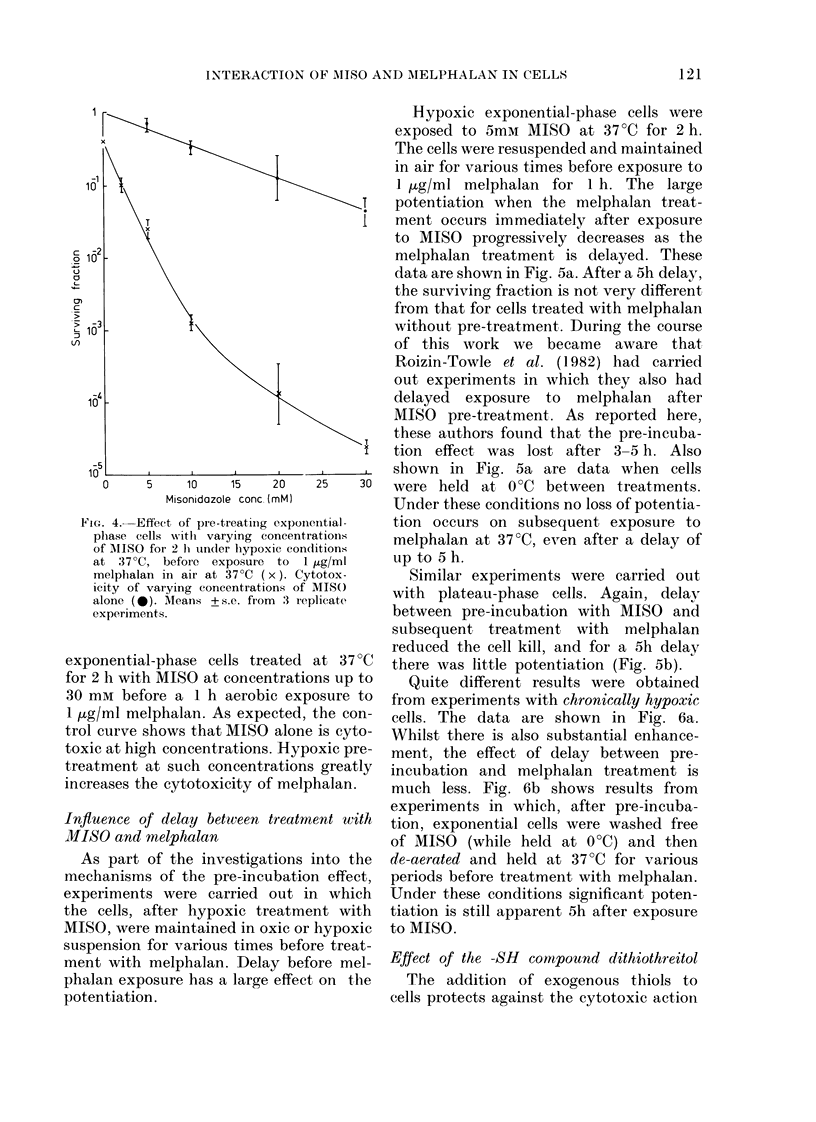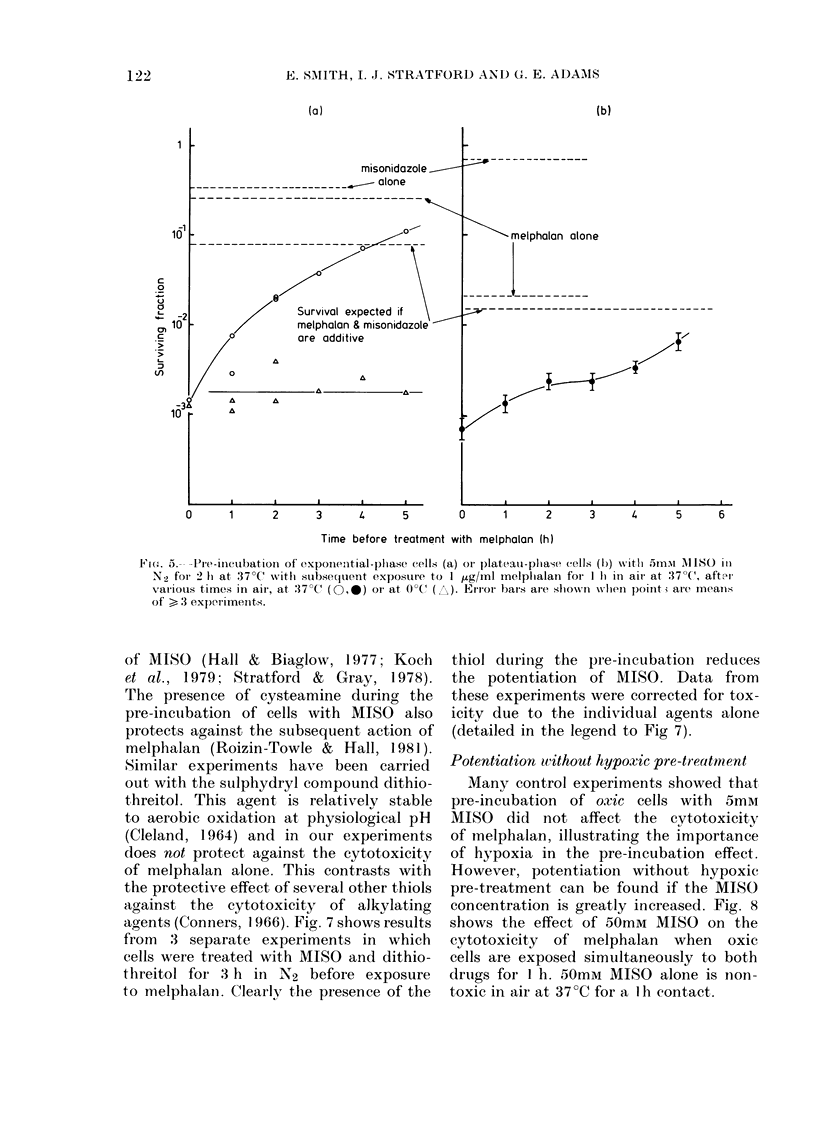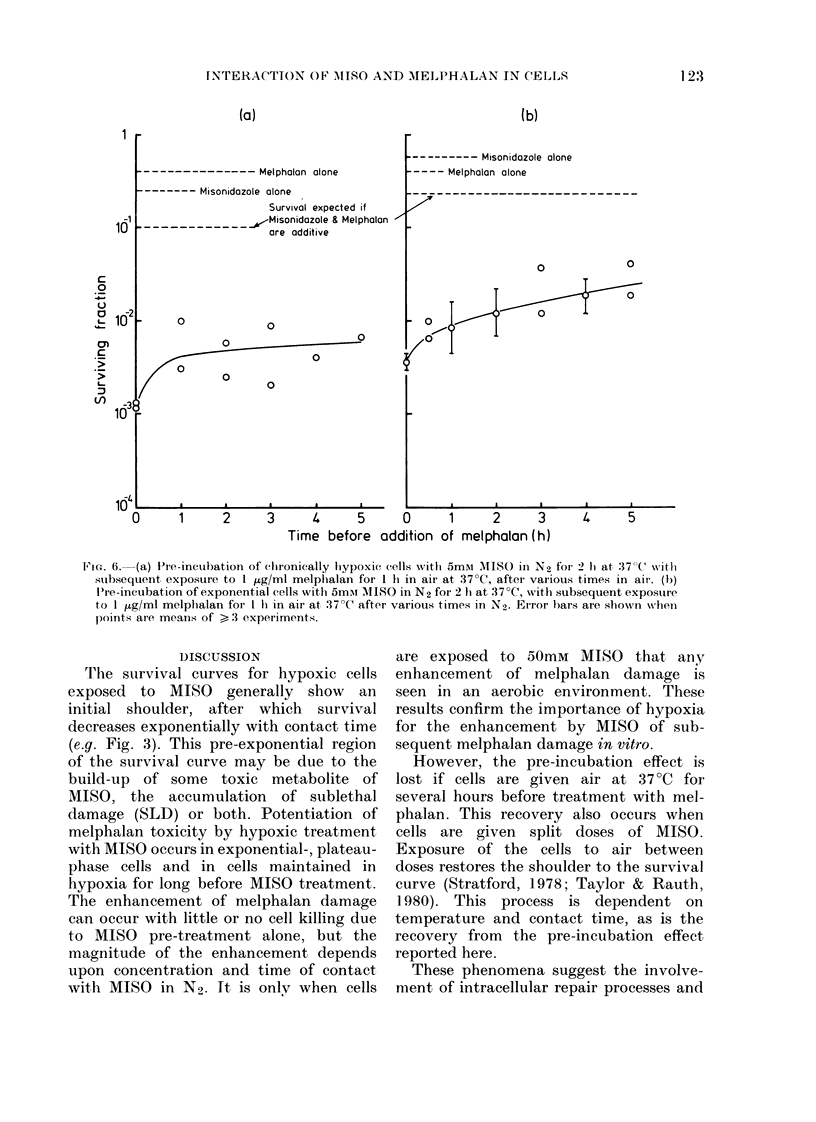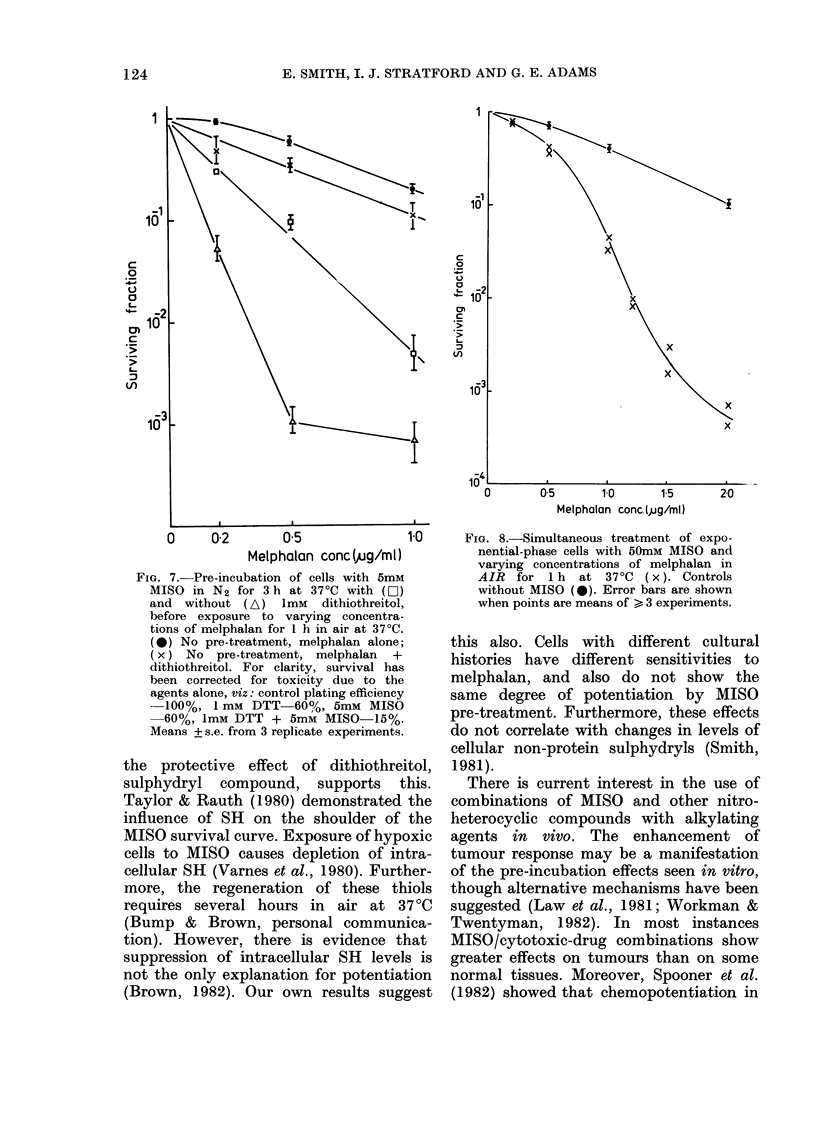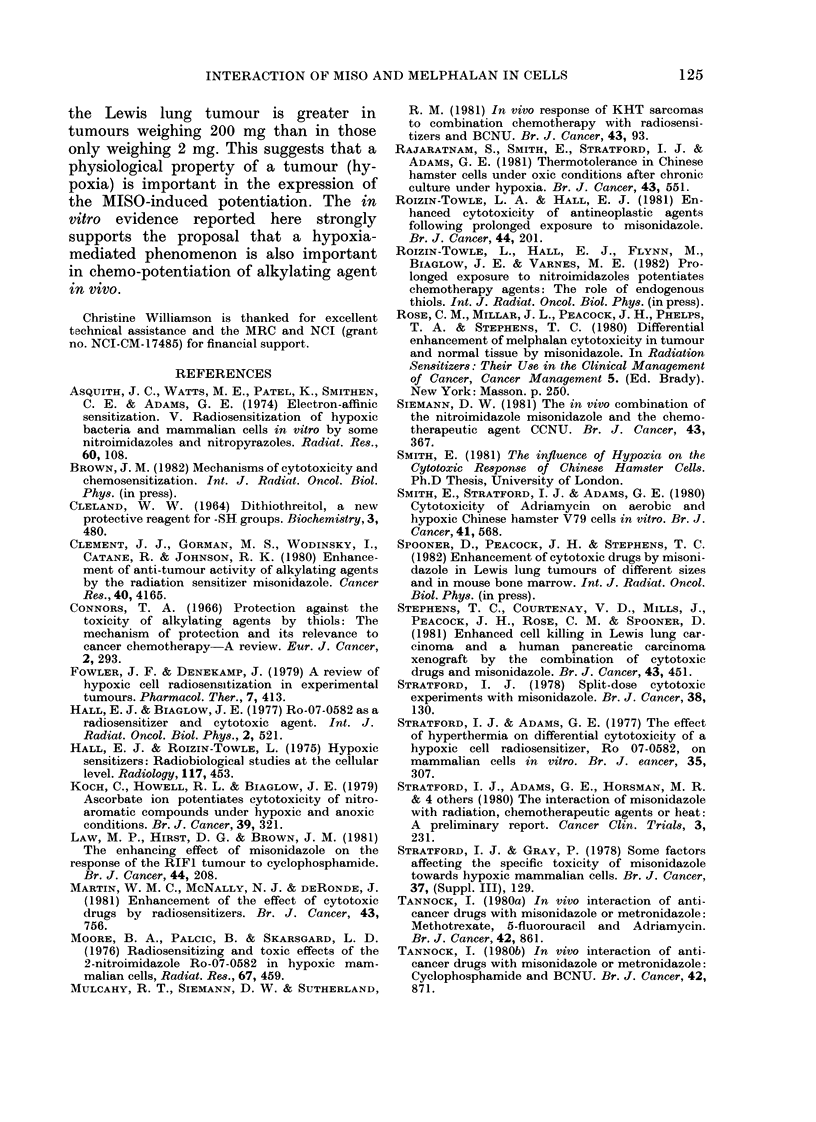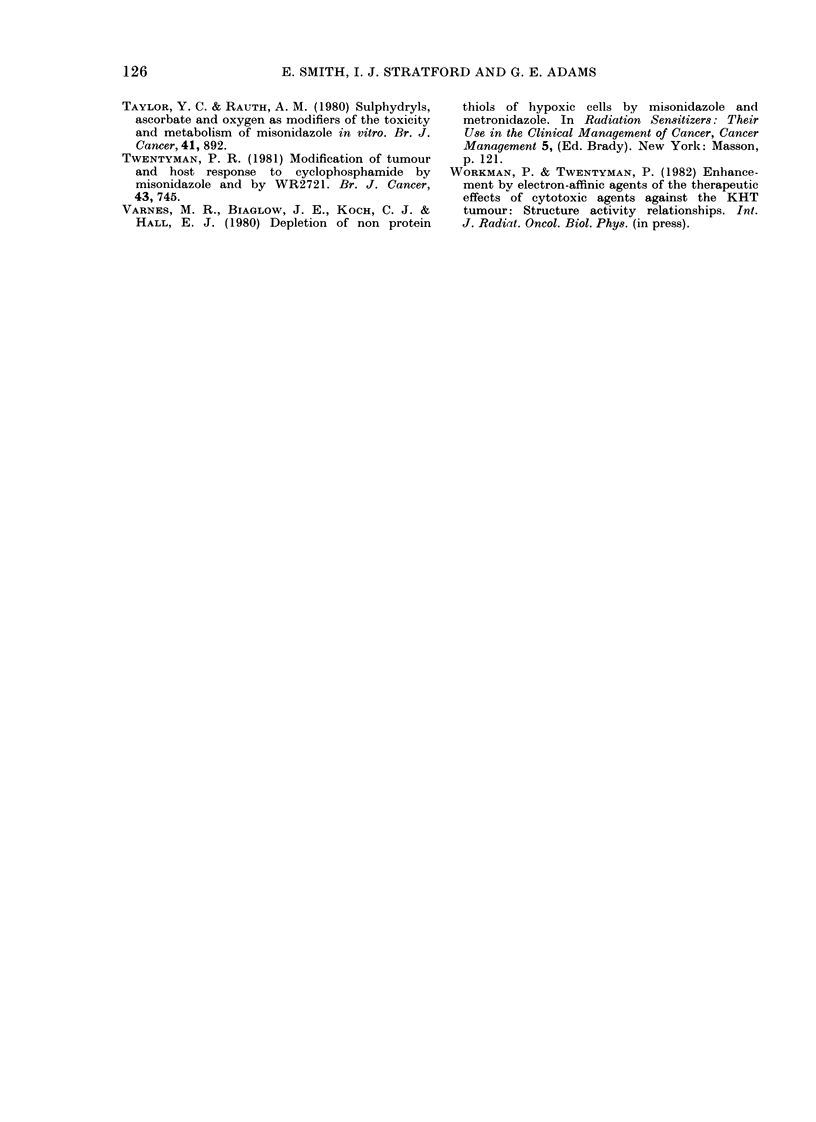# Enhancing effect of pre-treatment of cells with misonidazole in hypoxia on their response to melphalan in air.

**DOI:** 10.1038/bjc.1982.173

**Published:** 1982-07

**Authors:** E. Smith, I. J. Stratford, G. E. Adams

## Abstract

Pre-treatment of hypoxic cells with misonidazole (MISO) can render these cells more sensitive to a subsequent treatment with melphalan. Results in this paper show that this enhancement (or chemopotentiation) depends on the contact time and concentration of MISO, on the melphalan concentration and also on the cultural history of the cells. Damage due to hypoxic pre-incubation in MISO can be repaired if cells are subsequently aerated at 37 degrees C. In contrast, for cells washed free of MISO and then held under N2 at 37 degrees C, repair is much slower. No repair occurs when cells are held in air at 0 degrees C. The kinetics and extent of repair were dependent on the cells prior culture. Thus for exponential cells repair was complete after approximately 4 h, whereas for plateau-phase cells and cells with prior chronic hypoxia, repair was only partially complete after this time. Dithiothreitol was shown to protect partially against the enhancement of melphalan toxicity. Increased cell killing is also obtained if cells are given high concentrations of MISO (50 mM) in air during exposure to melphalan.


Br. J. Cancer (1982) 46, 117

### ENHANCING EFFECT OF PRE-TREATMENT OF CELLS WITH MISONIDAZOLE IN HYPOXIA ON THEIR RESPONSE TO MELPHALAN IN AIR

E. SMITH*, I. J. STRATFORD† AND G. E. ADAMS

*From the Radiobiology Group, Physics Division, F Block, Institute of Cancer Research, Sutton, Surrey SM2 5PX*

Received 14 December 1981 Accepted 19 February 1982

**Summary.—Pre-treatment of hypoxic cells with misonidazole (MISO) can render these cells more sensitive to a subsequent treatment with melphalan. Results in this paper show that this enhancement (or chemopotentiation) depends on the contact time and concentration of MISO, on the melphalan concentration and also on the cultural history of the cells. Damage due to hypoxic pre-incubation in MISO can be repaired if cells are subsequently aerated at 37°C. In contrast, for cells washed free of MISO and then held under $N_2$ at 37°C, repair is much slower. No repair occurs when cells are held in air at 0°C. The kinetics and extent of repair were dependent on the cells prior culture. Thus for exponential cells repair was complete after ~4 h, whereas for plateau-phase cells and cells with prior chronic hypoxia, repair was only partially complete after this time. Dithiothreitol was shown to protect partially against the enhancement of melphalan toxicity. Increased cell killing is also obtained if cells are given high concentrations of MISO (50 mM) in *air* during exposure to melphalan.**

MISONIDAZOLE (MISO) is an effective radiosensitizer of hypoxic cells *in vitro* and *in vivo* (Asquith *et al.*, 1974; Fowler & Denekamp, 1979) and is currently undergoing randomized, prospective clinical trials. It is also preferentially cytotoxic to hypoxic cells (Hall & Roizin-Towle, 1975; Moore *et al.*, 1976; Stratford & Adams, 1977).

It is known that MISO enhances anti-tumour activity of various alkylating agents *in vivo* (Rose *et al.*, 1980; Clement *et al.*, 1980; Tannock, 1980*a*,*b*; Law *et al.*, 1981; Martin *et al.*, 1981; Siemann, 1981; Mulcahy *et al.*, 1981; Stephens *et al.*, 1981; Twentyman, 1981). Enhancement of the cytotoxic action of melphalan also occurs *in vitro* (Stratford *et al.*, 1980; Roizin-Towle & Hall, 1981). Preliminary studies suggested that this *in vitro* effect operates at least partly through a hypoxia-mediated mechanism. If enhancement of response *in vivo* also involves, at least partly, hypoxia-dependent processes, it offers a prospect of obtaining selective enhancement of the response of tumours to cytotoxic drugs.

This paper reports an investigation of the hypothesis that hypoxia is necessary in the pre-treatment with MISO in order to increase the sensitivity to subsequent treatment with melphalan *in vitro*. The investigation also included a study of the influence of the cultural history of the cells, the duration of the hypoxic pre-treatment, the concentration of and duration of exposure to MISO on their subsequent sensitivity to various doses of melphalan.

### MATERIALS AND METHODS

*Cells.*—Chinese hamster V79-379A cells were grown in suspension, using methods described previously (Stratford & Adams,

* Present address: Richard Dimbleby Department of Cancer Research, St Thomas' Hospital Medical School, London SE1 7EH.

† To whom reprint requests should be made.

1977). Cells were maintained routinely in logarithmic phase at concentrations between $10^5$ and $10^6$/ml. The cell doubling time was 10–12 h, which required cells to be diluted daily.

For cells given a prolonged exposure to hypoxia, cultures were seeded at $5 \times 10^5$ ml. Cells in full growth medium were de-aerated in spinner flasks by passing $N_2$ continuously over the stirred suspension for 16 h (Smith *et al.*, 1980). The pH of the medium was maintained constant at 7·4 by $CO_2$/bicarbonate. Experiments showed that incubation in prolonged hypoxia under these conditions did not change the pH of the medium (Rajaratnam *et al.*, 1981).

Under the suspension-culture conditions, the cells attained plateau phase when their concentration reached $1{\cdot}8 \times 10^6$ ml (Stratford *et al.*, 1980). They were harvested from these unfed cultures 12 h later.

*Cytotoxicity experiments.*—Exponential-phase, plateau-phase and chronically hypoxic cells were treated in suspension in 250ml spinner flasks held in a water bath at 37 °C. MISO was added to the flasks and the cells deoxygenated by passing $N_2$ over the surface of the stirred suspension. At any given time under hypoxia, cells could be withdrawn for treatment with melphalan in air at 37 °C for 1 h. The cells were then centrifuged, resuspended, counted, diluted, plated in triplicate and incubated for 7 days at 37 °C, before scoring for colony formation.

*Compounds.*—MISO was provided by Roche Products Ltd, Welwyn Garden City, Herts. Melphalan was obtained from Burroughs Wellcome & Co. Ltd., London, and

FIG. 1.—Exponential-phase cells (a), or plateau-phase cells (b), treated with 5mM MISO for 2 h in hypoxia at 37 °C before exposure to varying concentrations of melphalan in air for 1 h at 37 °C (●). Cells treated with melphalan alone (○). Error bars are shown when points are means of ⩾3 experiments.

FIG. 2.—Chronically hypoxic cells treated with 5mM MISO for 2 h in hypoxia at 37°C before exposure to varying concentrations of melphalan in air for 1 h at 37°C (○). Cells treated with melphalan alone (●). Error bars are shown when points are means of ≥3 experiments.

was initially dissolved (1 mg/ml) in HCl/ethanol (2:98) before use. Dithiothreitol was supplied by Sigma Chemical Co., London.

### RESULTS

### *Chemopotentiation in exponential- and plateau-phase cells*

Experiments were carried out to compare the enhancing effects of MISO on the response to melphalan of both exponential- and plateau-phase cells. In both sets of experiments, the cells were exposed to 5mM MISO for 2 h under hypoxia at 37°C. The cells were then aerated and treated with various concentrations of melphalan for 1 h at 37°C.

Fig. 1a shows the results for exponential-phase cells. Treatment with MISO without subsequent exposure to melphalan causes a small reduction in the surviving fraction, to about 0·4. No correction for this small direct cytotoxicity of MISO has been made in calculating the surviving fractions in this figure. Controls were those cells, which before treatment with melphalan, were held in air with or without 5mM MISO or in hypoxia alone for 2 h. These treatments produced identical responses to melphalan. The linear proportions of the survival curves (fitted by eye) show that pre-treatment with MISO increases the slope about 4·5-fold. This is in general agreement with data published previously (Stratford *et al.*, 1980; Roizin-Towle & Hall, 1981.)

Fig. 1b shows data from similar experiments with plateau-phase cells. Potentiation also occurs, though in this case the enhancement ratio derived from the linear portions of the survival curves (2·5) is less than that for the exponential-phase cells. This difference appears to be due mainly to increased sensitivity of the control cells exposed to melphalan without prior MISO.

No potentiation occurs, either in exponential- or plateau-phase cells when the pre-treatment with MISO is under aerobic conditions.

### *Effect of prolonged hypoxia before pre-treatment with MISO*

Cells were maintained in hypoxia for 16 h and then, as described above, given 5mM MISO for a further 2 h in hypoxia, before treatment with melphalan for 1 h in air. Survival data are shown in Fig. 2. The control curve shows that chronic hypoxia slightly increases sensitivity to melphalan compared with acutely hypoxic cells and decreases it compared with plateau-phase cells. Thus, 2 µg/ml reduces survival to $10^{-1}$, $5 \times 10^{-2}$ and $1{\cdot}5 \times 10^{-3}$ for exponential, chronically hypoxic and plateau-phase cells respectively. However, although there is only a slight difference in sensitivity to melphalan between exponential and chronically hypoxic cells, the enhancement caused by MISO pretreatment is very

FIG. 3.—Pre-treatment of exponential-phase cells (a), or plateau-phase cells (b), with 5mM MISO for different times under hypoxic conditions at 37°C before exposure to 1 μg/ml melphalan in air at 37°C (×). Survival of cells given only 5mM MISO under hypoxic conditions (●). Error bars are shown when points are means of ≥3 experiments.

different: 4·5-fold and 2·5-fold, respectively.

*Pre-treatment with non-toxic concentrations of MISO*

The data in Figs 1 & 2 show that, even without subsequent melphalan treatment, a 2 h hypoxic exposure to 5mM MISO can cause slight cell killing. Experiments were carried out to determine whether potentiation of melphalan toxicity occurs when the pre-treatment with MISO killed no cells. Hypoxic cultures of either exponential- or plateau-phase cells were exposed to 5mM MISO for up to 5 h before treatment in air with 1 μg/ml of melphalan for 1 h. The data are shown in Fig. 3. The survival curves for cells treated with MISO alone show that in exponential-phase cells there is very little loss of viability for exposure times of less than 1½ h. However, even for these short exposure times, there is enhancement of melphalan toxicity. The surviving fraction of exponential-phase cells treated for 1 h with melphalan alone is 0·4 whereas pre-treatment with MISO for only 1 h before the melphalan exposure reduces survival to $4{\cdot}0 \times 10^{-2}$. In plateau-phase cells, melphalan alone is somewhat more effective, producing a surviving fraction of $7 \times 10^{-2}$. However, pretreatment with 5mM MISO for only 1 h under hypoxic conditions, which by itself causes no detectable cell kill, further increases melphalan toxicity by reducing survival to $3 \times 10^{-3}$.

*Effect of MISO concentration*

Fig. 4 shows survival data for hypoxic

FIG. 4.—Effect of pre-treating exponential-phase cells with varying concentrations of MISO for 2 h under hypoxic conditions at 37°C, before exposure to 1 μg/ml melphalan in air at 37°C (×). Cytotoxicity of varying concentrations of MISO alone (●). Means ±s.e. from 3 replicate experiments.

exponential-phase cells treated at 37°C for 2 h with MISO at concentrations up to 30 mM before a 1 h aerobic exposure to 1 μg/ml melphalan. As expected, the control curve shows that MISO alone is cytotoxic at high concentrations. Hypoxic pre-treatment at such concentrations greatly increases the cytotoxicity of melphalan.

*Influence of delay between treatment with MISO and melphalan*

As part of the investigations into the mechanisms of the pre-incubation effect, experiments were carried out in which the cells, after hypoxic treatment with MISO, were maintained in oxic or hypoxic suspension for various times before treatment with melphalan. Delay before melphalan exposure has a large effect on the potentiation.

Hypoxic exponential-phase cells were exposed to 5mM MISO at 37°C for 2 h. The cells were resuspended and maintained in air for various times before exposure to 1 μg/ml melphalan for 1 h. The large potentiation when the melphalan treatment occurs immediately after exposure to MISO progressively decreases as the melphalan treatment is delayed. These data are shown in Fig. 5a. After a 5h delay, the surviving fraction is not very different from that for cells treated with melphalan without pre-treatment. During the course of this work we became aware that Roizin-Towle *et al.* (1982) had carried out experiments in which they also had delayed exposure to melphalan after MISO pre-treatment. As reported here, these authors found that the pre-incubation effect was lost after 3–5 h. Also shown in Fig. 5a are data when cells were held at 0°C between treatments. Under these conditions no loss of potentiation occurs on subsequent exposure to melphalan at 37°C, even after a delay of up to 5 h.

Similar experiments were carried out with plateau-phase cells. Again, delay between pre-incubation with MISO and subsequent treatment with melphalan reduced the cell kill, and for a 5h delay there was little potentiation (Fig. 5b).

Quite different results were obtained from experiments with *chronically hypoxic* cells. The data are shown in Fig. 6a. Whilst there is also substantial enhancement, the effect of delay between pre-incubation and melphalan treatment is much less. Fig. 6b shows results from experiments in which, after pre-incubation, exponential cells were washed free of MISO (while held at 0°C) and then *de-aerated* and held at 37°C for various periods before treatment with melphalan. Under these conditions significant potentiation is still apparent 5h after exposure to MISO.

*Effect of the -SH compound dithiothreitol*

The addition of exogenous thiols to cells protects against the cytotoxic action

FIG. 5.—Pre-incubation of exponential-phase cells (a) or plateau-phase cells (b) with 5mM MISO in $N_2$ for 2 h at 37°C with subsequent exposure to 1 μg/ml melphalan for 1 h in air at 37°C, after various times in air, at 37°C (○,●) or at 0°C (△). Error bars are shown when points are means of ⩾3 experiments.

of MISO (Hall & Biaglow, 1977; Koch *et al.*, 1979; Stratford & Gray, 1978). The presence of cysteamine during the pre-incubation of cells with MISO also protects against the subsequent action of melphalan (Roizin-Towle & Hall, 1981). Similar experiments have been carried out with the sulphydryl compound dithiothreitol. This agent is relatively stable to aerobic oxidation at physiological pH (Cleland, 1964) and in our experiments does *not* protect against the cytotoxicity of melphalan alone. This contrasts with the protective effect of several other thiols against the cytotoxicity of alkylating agents (Conners, 1966). Fig. 7 shows results from 3 separate experiments in which cells were treated with MISO and dithiothreitol for 3 h in $N_2$ before exposure to melphalan. Clearly the presence of the thiol during the pre-incubation reduces the potentiation of MISO. Data from these experiments were corrected for toxicity due to the individual agents alone (detailed in the legend to Fig 7).

*Potentiation without hypoxic pre-treatment*

Many control experiments showed that pre-incubation of *oxic* cells with 5mM MISO did not affect the cytotoxicity of melphalan, illustrating the importance of hypoxia in the pre-incubation effect. However, potentiation without hypoxic pre-treatment can be found if the MISO concentration is greatly increased. Fig. 8 shows the effect of 50mM MISO on the cytotoxicity of melphalan when oxic cells are exposed simultaneously to both drugs for 1 h. 50mM MISO alone is non-toxic in air at 37°C for a 1h contact.

FIG. 6.—(a) Pre-incubation of chronically hypoxic cells with 5mM MISO in $N_2$ for 2 h at 37°C with subsequent exposure to 1 μg/ml melphalan for 1 h in air at 37°C, after various times in air. (b) Pre-incubation of exponential cells with 5mM MISO in $N_2$ for 2 h at 37°C, with subsequent exposure to 1 μg/ml melphalan for 1 h in air at 37°C after various times in $N_2$. Error bars are shown when points are means of ≥3 experiments.

### DISCUSSION

The survival curves for hypoxic cells exposed to MISO generally show an initial shoulder, after which survival decreases exponentially with contact time (*e.g.* Fig. 3). This pre-exponential region of the survival curve may be due to the build-up of some toxic metabolite of MISO, the accumulation of sublethal damage (SLD) or both. Potentiation of melphalan toxicity by hypoxic treatment with MISO occurs in exponential-, plateau-phase cells and in cells maintained in hypoxia for long before MISO treatment. The enhancement of melphalan damage can occur with little or no cell killing due to MISO pre-treatment alone, but the magnitude of the enhancement depends upon concentration and time of contact with MISO in $N_2$. It is only when cells are exposed to 50mM MISO that any enhancement of melphalan damage is seen in an aerobic environment. These results confirm the importance of hypoxia for the enhancement by MISO of subsequent melphalan damage *in vitro*.

However, the pre-incubation effect is lost if cells are given air at 37°C for several hours before treatment with melphalan. This recovery also occurs when cells are given split doses of MISO. Exposure of the cells to air between doses restores the shoulder to the survival curve (Stratford, 1978; Taylor & Rauth, 1980). This process is dependent on temperature and contact time, as is the recovery from the pre-incubation effect reported here.

These phenomena suggest the involvement of intracellular repair processes and

FIG. 7.—Pre-incubation of cells with 5mM MISO in $N_2$ for 3 h at 37°C with (□) and without (△) 1mM dithiothreitol, before exposure to varying concentrations of melphalan for 1 h in air at 37°C. (●) No pre-treatment, melphalan alone; (×) No pre-treatment, melphalan + dithiothreitol. For clarity, survival has been corrected for toxicity due to the agents alone, *viz:* control plating efficiency —100%, 1 mM DTT—60%, 5mM MISO —60%, 1mM DTT + 5mM MISO—15%. Means ±s.e. from 3 replicate experiments.

FIG. 8.—Simultaneous treatment of exponential-phase cells with 50mM MISO and varying concentrations of melphalan in *AIR* for 1 h at 37°C (×). Controls without MISO (●). Error bars are shown when points are means of ≥3 experiments.

the protective effect of dithiothreitol, sulphydryl compound, supports this. Taylor & Rauth (1980) demonstrated the influence of SH on the shoulder of the MISO survival curve. Exposure of hypoxic cells to MISO causes depletion of intracellular SH (Varnes *et al.*, 1980). Furthermore, the regeneration of these thiols requires several hours in air at 37°C (Bump & Brown, personal communication). However, there is evidence that suppression of intracellular SH levels is not the only explanation for potentiation (Brown, 1982). Our own results suggest this also. Cells with different cultural histories have different sensitivities to melphalan, and also do not show the same degree of potentiation by MISO pre-treatment. Furthermore, these effects do not correlate with changes in levels of cellular non-protein sulphydryls (Smith, 1981).

There is current interest in the use of combinations of MISO and other nitroheterocyclic compounds with alkylating agents *in vivo.* The enhancement of tumour response may be a manifestation of the pre-incubation effects seen *in vitro,* though alternative mechanisms have been suggested (Law *et al.*, 1981; Workman & Twentyman, 1982). In most instances MISO/cytotoxic-drug combinations show greater effects on tumours than on some normal tissues. Moreover, Spooner *et al.* (1982) showed that chemopotentiation in the Lewis lung tumour is greater in tumours weighing 200 mg than in those only weighing 2 mg. This suggests that a physiological property of a tumour (hypoxia) is important in the expression of the MISO-induced potentiation. The *in vitro* evidence reported here strongly supports the proposal that a hypoxia-mediated phenomenon is also important in chemo-potentiation of alkylating agent *in vivo*.

Christine Williamson is thanked for excellent technical assistance and the MRC and NCI (grant no. NCI-CM-17485) for financial support.

### REFERENCES

ASQUITH, J. C., WATTS, M. E., PATEL, K., SMITHEN, C. E. & ADAMS, G. E. (1974) Electron-affinic sensitization. V. Radiosensitization of hypoxic bacteria and mammalian cells *in vitro* by some nitroimidazoles and nitropyrazoles. *Radiat. Res.*, **60**, 108.

BROWN, J. M. (1982) Mechanisms of cytotoxicity and chemosensitization. *Int. J. Radiat. Oncol. Biol. Phys.* (in press).

CLELAND, W. W. (1964) Dithiothreitol, a new protective reagent for -SH groups. *Biochemistry*, **3**, 480.

CLEMENT, J. J., GORMAN, M. S., WODINSKY, I., CATANE, R. & JOHNSON, R. K. (1980) Enhancement of anti-tumour activity of alkylating agents by the radiation sensitizer misonidazole. *Cancer Res.*, **40**, 4165.

CONNORS, T. A. (1966) Protection against the toxicity of alkylating agents by thiols: The mechanism of protection and its relevance to cancer chemotherapy—A review. *Eur. J. Cancer*, **2**, 293.

FOWLER, J. F. & DENEKAMP, J. (1979) A review of hypoxic cell radiosensitization in experimental tumours. *Pharmacol. Ther.*, **7**, 413.

HALL, E. J. & BIAGLOW, J. E. (1977) Ro-07-0582 as a radiosensitizer and cytotoxic agent. *Int. J. Radiat. Oncol. Biol. Phys.*, **2**, 521.

HALL, E. J. & ROIZIN-TOWLE, L. (1975) Hypoxic sensitizers: Radiobiological studies at the cellular level. *Radiology*, **117**, 453.

KOCH, C., HOWELL, R. L. & BIAGLOW, J. E. (1979) Ascorbate ion potentiates cytotoxicity of nitroaromatic compounds under hypoxic and anoxic conditions. *Br. J. Cancer*, **39**, 321.

LAW, M. P., HIRST, D. G. & BROWN, J. M. (1981) The enhancing effect of misonidazole on the response of the RIF1 tumour to cyclophosphamide. *Br. J. Cancer*, **44**, 208.

MARTIN, W. M. C., MCNALLY, N. J. & DERONDE, J. (1981) Enhancement of the effect of cytotoxic drugs by radiosensitizers. *Br. J. Cancer*, **43**, 756.

MOORE, B. A., PALCIC, B. & SKARSGARD, L. D. (1976) Radiosensitizing and toxic effects of the 2-nitroimidazole Ro-07-0582 in hypoxic mammalian cells, *Radiat. Res.*, **67**, 459.

MULCAHY, R. T., SIEMANN, D. W. & SUTHERLAND, R. M. (1981) *In vivo* response of KHT sarcomas to combination chemotherapy with radiosensitizers and BCNU. *Br. J. Cancer*, **43**, 93.

RAJARATNAM, S., SMITH, E., STRATFORD, I. J. & ADAMS, G. E. (1981) Thermotolerance in Chinese hamster cells under oxic conditions after chronic culture under hypoxia. *Br. J. Cancer*, **43**, 551.

ROIZIN-TOWLE, L. A. & HALL, E. J. (1981) Enhanced cytotoxicity of antineoplastic agents following prolonged exposure to misonidazole. *Br. J. Cancer*, **44**, 201.

ROIZIN-TOWLE, L., HALL, E. J., FLYNN, M., BIAGLOW, J. E. & VARNES, M. E. (1982) Prolonged exposure to nitroimidazoles potentiates chemotherapy agents: The role of endogenous thiols. *Int. J. Radiat. Oncol. Biol. Phys.* (in press).

ROSE, C. M., MILLAR, J. L., PEACOCK, J. H., PHELPS, T. A. & STEPHENS, T. C. (1980) Differential enhancement of melphalan cytotoxicity in tumour and normal tissue by misonidazole. In *Radiation Sensitizers: Their Use in the Clinical Management of Cancer, Cancer Management* **5.** (Ed. Brady). New York: Masson. p. 250.

SIEMANN, D. W. (1981) The *in vivo* combination of the nitroimidazole misonidazole and the chemotherapeutic agent CCNU. *Br. J. Cancer*, **43**, 367.

SMITH, E. (1981) *The influence of Hypoxia on the Cytotoxic Response of Chinese Hamster Cells.* Ph.D Thesis, University of London.

SMITH, E., STRATFORD, I. J. & ADAMS, G. E. (1980) Cytotoxicity of Adriamycin on aerobic and hypoxic Chinese hamster V79 cells *in vitro*. *Br. J. Cancer*, **41**, 568.

SPOONER, D., PEACOCK, J. H. & STEPHENS, T. C. (1982) Enhancement of cytotoxic drugs by misonidazole in Lewis lung tumours of different sizes and in mouse bone marrow. *Int. J. Radiat. Oncol. Biol. Phys.* (in press).

STEPHENS, T. C., COURTENAY, V. D., MILLS, J., PEACOCK, J. H., ROSE, C. M. & SPOONER, D. (1981) Enhanced cell killing in Lewis lung carcinoma and a human pancreatic carcinoma xenograft by the combination of cytotoxic drugs and misonidazole. *Br. J. Cancer*, **43**, 451.

STRATFORD, I. J. (1978) Split-dose cytotoxic experiments with misonidazole. *Br. J. Cancer*, **38**, 130.

STRATFORD, I. J. & ADAMS, G. E. (1977) The effect of hyperthermia on differential cytotoxicity of a hypoxic cell radiosensitizer, Ro 07-0582, on mammalian cells *in vitro*. *Br. J. eancer*, **35**, 307.

STRATFORD, I. J., ADAMS, G. E., HORSMAN, M. R. & 4 others (1980) The interaction of misonidazole with radiation, chemotherapeutic agents or heat: A preliminary report. *Cancer Clin. Trials*, **3**, 231.

STRATFORD, I. J. & GRAY, P. (1978) Some factors affecting the specific toxicity of misonidazole towards hypoxic mammalian cells. *Br. J. Cancer*, **37**, (Suppl. III), 129.

TANNOCK, I. (1980*a*) *In vivo* interaction of anticancer drugs with misonidazole or metronidazole: Methotrexate, 5-fluorouracil and Adriamycin. *Br. J. Cancer*, **42**, 861.

TANNOCK, I. (1980*b*) *In vivo* interaction of anticancer drugs with misonidazole or metronidazole: Cyclophosphamide and BCNU. *Br. J. Cancer*, **42**, 871.

TAYLOR, Y. C. & RAUTH, A. M. (1980) Sulphydryls, ascorbate and oxygen as modifiers of the toxicity and metabolism of misonidazole *in vitro*. *Br. J. Cancer*, **41**, 892.

TWENTYMAN, P. R. (1981) Modification of tumour and host response to cyclophosphamide by misonidazole and by WR2721. *Br. J. Cancer*, **43**, 745.

VARNES, M. R., BIAGLOW, J. E., KOCH, C. J. & HALL, E. J. (1980) Depletion of non protein thiols of hypoxic cells by misonidazole and metronidazole. In *Radiation Sensitizers: Their Use in the Clinical Management of Cancer*, *Cancer Management* **5**, (Ed. Brady). New York: Masson, p. 121.

WORKMAN, P. & TWENTYMAN, P. (1982) Enhancement by electron-affinic agents of the therapeutic effects of cytotoxic agents against the KHT tumour: Structure activity relationships. *Int. J. Radiat. Oncol. Biol. Phys.* (in press).